# Energy replacement using glucose does not increase postprandial lipemia after moderate intensity exercise

**DOI:** 10.1186/1476-511X-13-177

**Published:** 2014-11-25

**Authors:** Chih-Hui Chiu, Stephen Francis Burns, Tsung-Jen Yang, Yi-Hsin Chang, Yi-Liang Chen, Cheng-Kang Chang, Ching-Lin Wu

**Affiliations:** Graduate Program in Department of Exercise Health Science, National Taiwan University of Sport, Taichung, Taiwan; Physical Education and Sports Science Academic Group, Nanyang Technological University, Singapore, Singapore; Department of Physical Education, National Taiwan Normal University, Taipei, Taiwan; Sport Science Research Center, National Taiwan University of Physical Education and Sport, Taichung, Taiwan; Graduate Institute of Sports Training, University of Taipei, Taipei, Taiwan; Graduate Institute of Sports and Health Management, National Chung Hsing University, Taichung, Taiwan

**Keywords:** Energy replacement, Postprandial lipemia, Energy deficit, LDL receptor

## Abstract

**Background:**

Aerobic exercise can decrease postprandial triglyceride (TG) concentrations but the relationship between exercise-induced energy deficits and postprandial lipemia is still unclear. The aim of the present study was to examine the effect of a single bout of aerobic exercise, with and without energy replacement, on postprandial lipemia and on peripheral blood mononuclear cell (PBMC) mRNA expression of very low density lipoprotein (VLDL) and low density lipoprotein (LDL) receptors and 3-hydroxy-3-methylglutaryl-CoA reductase (HMGCR).

**Methods:**

Nine healthy male humans completed three two-day trials in a random order. On day 1, volunteers rested (CON), completed 60 minutes of treadmill walking at 50% of VO_2peak_ (EX) or completed the same bout of walking but with the energy replaced afterwards with a glucose solution (EXG). On day 2, volunteers rested and consumed a high fat test meal in the morning.

**Results:**

Total and incremental TG AUC were significantly lower on the EXG (*P* < 0.05) and EX (*P* < 0.05) trials than the CON trial with no difference between the two exercise trials. No significant difference was observed in VLDL or LDL receptor mRNA expression among the trials (*P* > 0.05).

**Conclusions:**

In conclusion, energy replacement by glucose did not affect the decrease in postprandial TG concentrations observed after moderate intensity exercise and exercise does not affect changes in PBMC HMGCR, VLDL and LDL receptor mRNA expression.

## Background

Prospective cohort studies demonstrate a strong link between non-fasting triglyceride (TG) concentrations and cardiovascular disease or mortality in both men and women [1–3]. Moreover, increased postprandial TG concentrations also appear to promote blood remnant cholesterol [4], thereby indirectly contributing to atherosclerosis [5]. It has been shown that a single bout of aerobic exercise can decrease postprandial TG concentrations [6–8]. The energy expenditure of exercise is postulated to be a more important determinant behind reductions in postprandial lipemia than exercise duration or intensity [7, 9, 10]. Importantly for public health, this means moderate intensity exercise such as brisk walking is effective at reducing postprandial TG concentrations whether accumulated in short bouts throughout the day or a single longer bout as long as the energy expenditure of exercise is sufficient [8].

Whilst the energy expenditure of aerobic exercise has been demonstrated to be related to the reduction in postprandial lipaemia [7] the relationship between energy deficits and reductions in postprandial TG concentrations is not clearly defined [11]. An exercise-induced energy deficit results in a greater reduction in postprandial TG concentration than a dietary-induced energy deficit [12]. Conversely, post-exercise restoration of an exercise-induced energy deficit dramatically attenuates any decrease in postprandial TG concentrations the next day [13, 14]. To replace the energy debt in one of these studies, an oral supplementation of glucose was given immediately, 2 and 4 hours post vigorous exhaustive exercise which significantly depleted muscle glycogen reserves and oxidized only a small amount of fat [14]. However, it is unclear whether the attenuated response in these studies was because of replacement of the energy deficit created by exercise or replacement of the substrate utilized during exercise. Harrison et al. suggest that an oral glucose supplementation given after intense, exhaustive exercise was important in the attenuated TG response the next day as high carbohydrate diets induce hypertriglyceridemia [14]. However, exaggeration of postprandial lipemia [15] by carbohydrate is associated with fructose and not glucose intake [16]. The possibility exists, therefore, that had fat oxidation been higher during exercise then glucose re-feeding would not have reversed the reduction seen in postprandial lipemia as intramuscular TG would not have been replaced. Therefore, had fat oxidation been high during exercise then glucose re-feeding might not have reversed the reduction seen in postprandial lipemia. Certainly, there were no studies have investigated the fat to carbohydrate deficit on postprandial lipemia.

A number of studies have shown that TG-rich lipoproteins, chylomicrons and very-low-density lipoproteins (VLDL), can be bound and also directly internalized by cells via the VLDL receptor, a member of the low-density lipoprotein (LDL) receptor family [17, 18]. The VLDL receptor is widely expressed on the capillary endothelium of skeletal muscle and adipose tissue but only in trace amounts in the liver [17]. Conversely, the LDL receptor is abundantly expressed in liver [17] and internalization of LDL particles reduces cellular expression of 3-hydroxy-3-methylglutaryl-CoA reductase (HMGCR), an important enzyme involved in cellular cholesterol synthesis [17]. The effect of acute aerobic exercise, with and without energy replacement, on the expression of liver VLDL and LDL receptor and HMGCR has not received attention. However, prior exercise can reduce the postprandial concentration of remnant lipoprotein particle cholesterol and TG [19, 20]. Given the role of the VLDL and LDL receptors in the uptake of TG-rich lipoprotein remnant particles it seems logical that hepatic expression of these receptors might change in the postprandial period in response to exercise. Any change in cholesterol uptake by the liver would also affect liver expression of HMGCR. A method to study liver lipid related mRNA expression via peripheral blood samples was proposed by Powell and Kroon [21]. Studies have shown that the expression of these mRNA in peripheral blood mononuclear cell could reflect parallel their action in liver [21, 22]. Analyses of mRNA expression in peripheral blood mononuclear cell have been demonstrated in previous studies [22, 23].

Thus, the aims of the present study were two-fold: i) to examine the effect of using carbohydrate to restore an energy deficit induced by moderate intensity exercise on postprandial lipemia and ii) to examine changes in the mRNA expression of PBMC VLDL and LDL receptor and PMBC HMGCR, potentially relevant to remnant particle clearance.

## Results

### Treadmill walking

The self selected walking speed during the EXG and EX trial were 6.0 ± 0.1 km/h with 8.0 ± 2.0% of inclination. The energy expenditure at 50% VO_2peak_ for 60 min was calculated by a regression equation from the result of the 16-min submaximal oxygen uptake test, was 520 ± 50.4 kcal. The glucose replacement during EXG trial was 130.4 ± 12.6 g. The substrate oxidations during the exercise estimated from the submaximal test were 9.9 ± 2.6 g from fat and 116.1 ± 13.7 g from carbohydrate.

### Dietary information on day 1

The total energy consumed day 1 was 2052 ± 64.2 kcal with 237.1 ± 15.6 g of carbohydrate (46.40 ± 0.03%), 79.1 ± 3.9 g of fat (34.70 ± 0.01%) and 99.8 ± 15.3 g of protein (19.40 ± 0.03%). The breakfast contained 525.9 ± 14.3 kcal, with 45.8 ± 1.2% energy from carbohydrate (60.3 ± 2.8 g), 41.1 ± 1.3% from fat (24.1 ± 1.1 g), and 13.0 ± 1.5% from protein (17.1 ± 0.6 g). The lunch provided 840.0 ± 57.0 kcal, with 50.7 ± 0.3% energy from carbohydrate (106.5 ± 7.4 g), 31.5 ± 0.5% from fat (29.4 ± 1.8 g), and 17.8 ± 0.5% from protein (37.5 ± 3.2 g). The standard dinner offered 692 kcal, with 50% energy from carbohydrate (86.5 g), 32% from fat (24.6 g), and 18% from protein (31.1 g).

### Fasting plasma concentrations

Fasting concentrations of all measured plasma metabolites were similar on the morning of day 2 in all trials (Table 1).

**Table 1:** **Plasma concentrations in the fasted state on the morning of day 2 of each main trial**

	EXG	EX	CON	***P***value
TG (mmol/L)	0.68 ± 0.08	0.89 ± 0.19	0.93 ± 0.09	0.223
TC (mmol/L)	4.13 ± 0.25	4.65 ± 0.23	4.58 ± 0.25	0.153
HDL-C (mmol/L)	1.57 ± 0.08	1.74 ± 0.13	1.78 ± 0.16	0.189
LDL-C (mmol/L)	2.30 ± 0.26	2.57 ± 0.21	2.44 ± 0.21	0.348
Glucose (mmol/L)	4.59 ± 0.30	4.72 ± 0.16	4.91 ± 0.11	0.320
Insulin (pmol/L)	48.31 ± 10.36	49.50 ± 7.39	58.24 ± 5.88	0.315
NEFA (mmol/L)	0.51 ± 0.04	0.57 ± 0.06	0.55 ± 0.09	0.479
Glycerol (μmol/L)	52.56 ± 5.83	61.33 ± 5.72	51.00 ± 5.04	0.088
3-HB (μmol/L)	41.11 ± 10.80	58.89 ± 16.73	28.44 ± 8.31	0.084

Values are mean SEM, n = 9. CON, control; EXG, exercise with glucose replacement; EX, exercise only; TG, triglyceride; TC, total cholesterol; HDL-C, high-density lipoprotein-cholesterol; LDL-C, low density lipoprotein-cholesterol; NEFA, Non-esterified fatty acids; 3-HB, 3-hydroxybutyrate.

### Postprandial plasma concentrations

**Figure 1:**
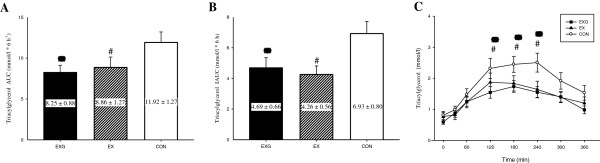
**Total (A) and incremental (B) 6 hour area under the triglyceride (TG) concentration versus time curve and fasting and postprandial TG concentrations over the 6 hours (C) on the control (CON) (○),exercise (EX) (**▲**) and exercise with glucose replacement (EXG) (■) trials.** Values are mean ± SEM, *n* = 9. *EXG group significantly different from CON (*P* < 0.05). #EX group significantly different from CON (*P* < 0.05).

Total (Figure 1A) and incremental (Figure 1B) TG AUC were significantly lower on the EXG (31% for AUC and 32% for IAUC, both *P* < 0.05) and EX (26% for AUC and 39% for IAUC, both *P* < 0.05) trial than the CON trial. No significant differences were observed between the EXG and EX trials in total and incremental AUC. Plasma TG responses over the six hours (Figure 1C) were lower on both exercise trials than on the control trial with no difference between the EXG and EX trial (main effect of trial, *P* = 0.001). There was a significant trial by time interaction (*P* = 0.009) with TG concentrations significantly higher between 2 and 4 hours on the Con trial compared with the two exercise trials.

**Figure 2:**
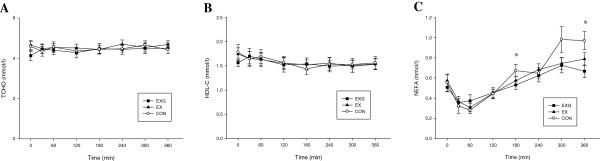
**Fasting and postprandial 6 hour concentrations of total cholesterol (TCHO) (A), high density lipoprotein cholesterol (HDL-C) (B) and Non-esterified fatty acids (NEFA) (C) on the control (CON) (○), exercise (EX) (**▲**) and exercise with glucose replacement (EXG) (■) trials**. Values are mean ± SEM, *n* = 9. *EXG group significantly different from CON (*P* < 0.05).

Plasma concentrations of TC, HDL-C and NEFA are shown in Figure 2. There were no differences among the three trials in concentrations of TC (Figure 2A), HDL-C (Figure 2B) or NEFA (Figure 2C). The TC concentrations did not change over the morning but HDL-C fell slightly over the six hours on all trials (main effect of time, *P* < 0.001). There was a significant interaction between groups and times (p = 0.001). Plasma NEFA concentrations were significant lower in EXG compared to CON at 180 and 360 min.

Summary postprandial responses for plasma insulin, glucose, glycerol, NEFA and 3-HB are provided in Table 2. No significant differences were observed in plasma insulin, glucose, glycerol or 3-HB among trials. Insulin and glucose concentrations peaked in the first hour after the meal and then fell steadily to baseline concentrations throughout the 6 hour period on all three trials. Conversely, glycerol and 3-HB fell rapidly in the first 1–2 hours after the test meal and then increased throughout the rest of the 6 hours on all trials. There was a significant trial by time interaction for plasma NEFA concentrations (*P* = 0.001) (Figure 2). Plasma NEFA concentrations fell rapidly in the first hour after consumption of the test meal on all trials and then increased until the end of the 6 hours. The NEFA concentrations were significantly decreased in the EXG trial at 180 and 360 min postprandial compared with CON. There was no significant difference in NEFA concentrations between the EX and CON trials or between the two exercise trials (Figure 2C).

**Table 2:** **The postprandial responses for plasma insulin, glucose, glycerol, FFA and 3-HB**

	EXG	EX	CON	***P***value
Glucose (mmol/L*6 h ^*−1*^ *)*				
Total AUC	29.97 ± 1.72	29.83 ± 1.08	30.04 ± 1.16	0.972
Incremental AUC	3.14 ± 1.10	2.08 ± 0.51	1.57 ± 0.46	0.246
Insulin (pmol/L*6 h ^*−1*^ *)*				
Total AUC	920.12 ± 194.16	782.55 ± 94.03	976.27 ± 114.28	0.270
Incremental AUC	635.42 ± 143.74	509.06 ± 69.10	631.86 ± 95.58	0.433
NEFA (mmol/L*6 h ^*−1*^ *)*				
Total AUC	3.26 ± 0.22	3.40 ± 0.23	3.79 ± 0.30	0.119
Glycerol (μmol/L*6 h ^*−1*^ *)*				
Total AUC	344.97 ± 18.53	353.78 ± 21.34	360.75 ± 23.00	0.996
3-HB (μmol/L*6 h ^*−1*^ *)*				
Total AUC	312.26 ± 84.13	545.13 ± 199.83	246.94 ± 57.08	0.226

Values are mean SEM, n = 9. CON, control; EXG, exercise with glucose replacement; EX, exercise only; NEFA, Non-esterified fatty acids; 3-HB, 3-hydroxybutyrate.

### Receptor mRNA expression

**Figure 3:**
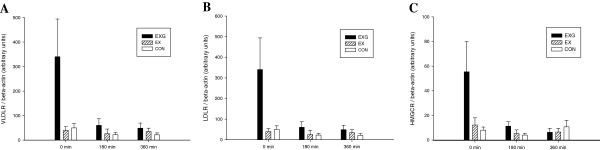
**Fasting and postprandial 6 hour mRNA expression of the PBMC very low density lipoprotein (VLDL) receptor (A), low density lipoprotein (LDL) receptor (B) and 3-hydroxy-3-methylglutaryl-CoA reductase (HMGCR)** (C) **on the control (CON)** (□)**, exercise (EX) (▨**) **and exercise with glucose replacement (EXG) (■) trials.** Values are mean ± SEM, *n* = 9.

Expression of VLDL receptor, LDL receptor, and HMGCR mRNA are shown in Figure 3. No significant difference was observed in mRNA expression among the trials although the mRNA expression of the LDL receptor (*P* = 0.056) and HMGCR (*P* = 0.039) changed over time (main effect for time). There were no interaction effects for the mRNA expression of VLDL, LDL receptors and HMGCR.

## Discussion

The present study is the first to demonstrate that aerobic exercise followed by immediate post-exercise glucose replacement can reduce postprandial plasma TG concentrations the next morning as effectively as exercise without energy replacement. Our data contrast with two previous studies which found that post-exercise energy replacement mitigated any benefit on postprandial TG concentrations the next day [13, 14]. Given that we used moderate intensity exercise with a glucose energy replacement in the present study suggests that substrate replacement may not play an important role in determining the effect of any exercise bout on postprandial lipemia. Our results implicated that fat deficit induced by aerobic exercise may improve the postprandial TG response the next morning.

Two previous studies using aerobic exercise found that energy replacement dramatically attenuated the effect of exercise on postprandial lipemia [13, 14]. The first of these studies found that a meal-replacement drink containing 48% carbohydrate, 38% fat and 14% protein attenuated the effects on postprandial lipemia the morning after a treadmill walk at 50% VO_2peak_ expending 27 kJ/kg body mass [13]. The second study used glucose, given intermittently over 4 hours, to replace the energy used in a 90 minute cycling bout at 70% VO_2peak_ which was followed by ten 1-minute sprints and which expended ~1508 kcal [14]. These two studies contrast with our findings with the major difference being that the glucose energy replacement we used was unlikely to replace the substrate used during exercise. Exercise at 50% VO_2peak_ is nearing the maximal rate of fat oxidation for many individuals [24, 25] and the glucose solution we used would not have replaced the fat oxidized during exercise.

The relative importance of a fat deficit from exercise on postprandial lipemia has received little attention. We believe the only study to have examined this issue directly compared the effect of two 90 minute runs at 60% VO_2max_ with or without acipimox to inhibit lipolysis from adipose tissue during exercise [26]. In that study both exercise trials were equally effective in ameliorating postprandial TG concentrations in response to a high fat meal taken the next morning. The authors suggested that the magnitude of fat metabolism during exercise was not the major determinant of the effect of exercise on postprandial lipemia. However, it should be noted that study fat oxidation still contributed 16% of the energy expenditure to exercise even with acipimox and the authors suggest that acipimox use probably did not inhibit intramuscular TG breakdown. On the other hand, acipimox also increases oxidation of carbohydrate as energy sources, while the role of carbohydrate oxidation during exercise on postprandial TG response has not been concluded from this study. Given that a glucose drink replacement failed to mitigate the effects of exercise on postprandial lipemia in the present study, we suggest our findings support a hypothesis that fat oxidation does play some role in determining post-exercise reductions in postprandial lipemia. However, we note that we did not measure muscle TG uptake or intramuscular TG concentrations before or after exercise so direct evidence for this hypothesis is lacking.

The role of carbohydrate in mediating the effects of exercise on postprandial lipemia has been suggested to be important. Harrison and colleagues [14] suggest that glucose re-feeding in their study and the replacement of muscle glycogen after intense exercise may indicate the importance of a carbohydrate over a fat deficit in mediating the exercise effects on postprandial metabolism. This is supported by studies demonstrating that short-term carbohydrate diets exaggerate postprandial lipemia [19, 27]. However, it is well documented that the effect of carbohydrate on TG concentrations is primarily associated with fructose (or sucrose) intake and not glucose [16, 28]. Our data support this as the glucose energy replacement solution we provided had no effect on fasting or postprandial TG the next morning despite the fact that the exercise intensity was only 50% VO_2peak_ and total energy expenditure of the exercise bout was only ~1/3 of that in the study by Harrison and colleagues. In addition, the timing of energy replacement in present study was 2 hour after exercise, which may not be ideal nutritional supplementation timing for muscle glycogen recovery. The different exercise intensity, total energy expenditure of the exercise and energy replacement timing after exercise in this study may result in lower muscle glycogen concentration when compared to Harrison and colleagues. We suggest that the attenuated lipemia seen in the study by Harrison and colleagues was a result of energy replacement and replacement of glycogen stores used by the muscle during exercise rather than a relative importance of a carbohydrate over a fat deficit.

To our knowledge no study has investigated the effect of acute exercise on postprandial PBMC VLDL or LDL receptor mRNA expression after a high fat meal. A single bout of exercise is known to reduce the postprandial concentration of TG-rich remnant lipoprotein particle cholesterol and TG [19, 20]. Thus, we hypothesized increased expression of the PBMC VLDL and LDL receptor and a down-regulation of HMGCR mRNA in the liver after the exercise bouts in the present study but we found no differences among trials. The VLDL receptor is poorly expressed in liver and no change in PBMC expression in the present study likely reflects that other pathways – decreased hepatic VLDL secretion or increased muscle TG uptake – are more important in contributing to reduced remnant lipoprotein TG after exercise. Similarly, we found no differences among trials in LDL receptor mRNA expression even though expression of PBMC HMGCR mRNA did change over time after feeding, suggesting reduced endogenous hepatic cholesterol synthesis. Although we did not measure remnant lipoprotein cholesterol concentrations, which might be one of the limitation of current study, the remnant lipoprotein cholesterol concentration has been shown to predict carotid artery intima media thickness [29] and remnant lipoproteins have been identified as a particularly atherogenic subclass of lipoproteins [4, 5]. However, remnant lipoprotein cholesterol contribution to the total cholesterol pool is small compared with total LDL cholesterol [20] which likely explains why PBMC LDL receptor mRNA expression was not increased after exercise.

We acknowledge several limitations to the present study. One of the major limitations of this study is that we did not measure substrate oxidation during the exercise bout as participants were unable to keep the mouthpiece in for the duration of the walk. Nonetheless, according to our submaximal exercise test, the estimated substrate oxidation was 9.9 ± 2.6 g from fat and 116.1 ± 13.7 g from carbohydrate for 60 min of 50% VO_2peak_ walking which is not intense exercise. Harrison and colleagues directly measured participants muscle glycogen concentrations before and after exercise using muscle biopsy but we did not have access to this technique and could not, therefore, examine changes in muscle substrate concentrations after the exercise bouts. Indeed, intramyocellular triacylglycerol concentration has been shown to decrease for 62.7% after moderate endurance exercise [30]. We hypothesize that intramuscular TG deficit is important in reducing postprandial lipemia but biopsy studies to determine muscle substrate concentrations along with studies on TG extraction across the muscle after exercise are needed to determine the effects of energy replacement on postprandial lipemia [31].

In conclusion, the present study found that energy replacement by glucose did not affect the decrease in postprandial TG concentrations observed after moderate intensity exercise where fat oxidation contributes most to energy expenditure. Future studies should closely examine the role of substrate oxidation during exercise on postprandial lipemia. We also found no change in PBMC HMGCR, VLDL or LDL receptor changes after exercise with or without energy replacement and suggest that other pathways are of more importance in reducing remnant lipoprotein TG and cholesterol than the expression of these receptors.

## Methods

### Subjects

Nine healthy, untrained and active males (mean ± SEM; age: 23.8 ± 0.2 years, height: 1.72 ± 0.02 m, body mass: 69.8 ± 1.5 kg, VO2peak: 49.0 ± 3.9 ml/kg/min) volunteered to complete this study after approval by the Human Subject Committee of National Taiwan College of Physical Education. All participants gave their written informed consent after an explanation of the procedures and risks involved. Participants were screened for any potential health issues by questionnaire before testing. No participants were taking any medication.

### Experimental design

Participants completed three two-day trials. During day one of each trial, the participants either 1) remained sedentary (CON), 2) performed 60 min of treadmill walking at 50% of peak oxygen uptake (EX), or 3) performed 60 min of treadmill walking at 50% of peak oxygen uptake with the energy expended from exercise replaced by glucose (EXG). On day two of each trial, the participants consumed a high fat test meal in the morning. Each trial was performed in a random order. Participants were asked to refrain from caffeine and alcohol for 1 week before all main trials and instructed to avoid vigorous physical activity for 3 days before all main trials. All subjects were asked to record 3 days diet before the first main trial, and were asked to repeat the same diet 3 days before the other trials.

### Preliminary tests

At least 7 days before main trials began, participants were asked to perform two preliminary tests on a treadmill (Medtrack ST65, Quinton, Seattle, Washington, USA) for calculated the relationship between O_2_ uptake, submaximal walking inclination and energy expenditure:

- *submaximal oxygen uptake test:* A 16 min, continuous submaximal walking test, consisting of four stages, was used to determine the relationship between submaximal walking inclination and oxygen uptake. The speed of the treadmill was self-selected by participants between 5.5 and 6.5 km/h. Treadmill inclination was increased from an initial 0% by 2.5% every 4 minutes. At the end of the test participants were given 30 minutes to recover before they completed a peak oxygen uptake (VO_2peak_) test.
- *Peak oxygen uptake test:* Participants completed an uphill treadmill walking test, at a constant speed until they reached volitional fatigue, in order to determine their VO_2peak_. The speed of the treadmill was set at 6.0 to 7.0 km/h depending on each participant’s fitness level. The inclination was increased from an initial 0% by 2.5% every 3 minutes until subjects reached volitional fatigue.

Expired air samples were determined during both tests with the use of gas analyzer (Vmax Series 29C, Sensor Medics, California, USA). A regression equation was used to calculate the relationship between VO_2_ and walking inclination. The energy expenditure for 60 min walking at 50% VO_2peak_ was calculated from the result of the 16-min submaximal oxygen uptake test.

### Main trials

#### Day 1

On the first day of each main trial, subjects were asked to consume breakfast and lunch by themselves at 0800–0900 and 1200–1300, respectively. All subjects were asked to visit the laboratory at 1600. During the two exercise trials participants performed 60 minutes of treadmill (Medtrack ST65, Quinton, Seattle, Washington, USA) walking at 50% VO_2peak_, and stopped exercising at ~1700. All participants were asked to rest for at least 2 hours in the laboratory post-exercise. Participants were allowed to drink water *ad libitum* during this recovery period. A dinner was provided by a dietitian at ~1900. The energy provided by the dinner was 692 kcal, with 50% energy from carbohydrate, 32% from fat, and 18% from protein. On the EXG trial a weighted glucose solution dissolved in water was provided with the dinner. The energy in this solution was equivalent to the energy expended during the prior exercise bout. On the CON trial, participants rested in the laboratory from 1600 until dinner was provided at the same time as on the exercise trials. At the end of the meal participants returned home to rest. They were not allowed to consume any other food or drinks except water until they returned to the laboratory the next morning.

#### Day 2

The next morning participants reported to the laboratory at 0800. Participants sat in the laboratory for 10 min after which a fasting blood sample was collected by a cannula inserted into an antecubital vein. A 10 mL sterile sodium chloride solution (0.9% w/v) was used to flush and clean the cannula after each blood sampling. Participants then consumed a high fat test meal for breakfast. Participants were asked to consume the meal within 20 min. A timer was started immediately after subjects consumed the test meal. Further blood samples were collected at 0.5, 1, 2, 3, 4, 5 and 6 hours after the end of the meal. Peripheral blood mononuclear cells mRNA was extracted from the fasting sample and at 3 and 6 hours. The subjects were asked to remain seated and resting in the laboratory until the end of the six hour testing period. Participants were allowed to drink water *ad libitum* during the first trial. The timing and volume of water intake were recorded on the first trial and repeated in the following two trials.

### High fat test meal

The test meal consisted of whipping cream, butter, cereal, nuts, and white bread. The test meal was prescribed according to body mass and provided 1.2 g fat, 1.1 g carbohydrate, 0.33 g protein, and 69.3 kJ/kg of body mass. Macronutrient contents of the meal were based on those provided by the manufacturers of the foods.

### Blood sample collection

A cannula (Venflon 20G, Sweden) connected to a 3-way stopcock (Connecta Ltd., Sweden) with a 10 cm extension tube was used to collect 10 mL venous blood samples at each time point. The blood samples were collected into EDTA tubes and blood cell counts immediately measured by a cell counter (Sysmax KX-21 N, Kobe, Japan) and the concentration of hemoglobin and hematocrit used to calculate any changes in plasma volume using accepted formula [32]. The remaining blood sample was then centrifuged at 500 g’ (Eppendorf 5810, Hamburg, Germany) for 20 mins to extract the plasma. The plasma was aliquoted and stored at −70°C for analysis at a later date.

### Blood analytical methods

Plasma concentrations of TG, non-esterified fatty acids (NEFA), glucose, glycerol, total cholesterol (TC), high density lipoprotein-cholesterol (HDL-C) and 3-hydroxybutyrate (3-HB) were measured with an automated analyzer (Hitachi 7020, Tokyo, Japan) using commercially available kits (Randox, Antrim, UK). Plasma concentrations of insulin were measured by electrochemiluminescence (Elecsys 2010, Roche Diagnostics, Basel, Switzerland) using a commercial kit.

### mRNA quantification

#### Isolation mononuclear cells and RNA preparation

Peripheral blood mononuclear cells were harvested using the ficoll separation method [33]. After the plasma was removed from the centrifuged EDTA sample, the white blood cells were removed immediately from the buffy coat. Then 6 ml of HISTOPAQUE® -1077 (Sigma-Aldrich) were put into the removed white blood cells for 15 min and centrifuged at 1800 rpm for 3 min. The supernatant was removed and diluted by 10 mL of buffer A (10 mM Phosphate buffer, pH 7.4, 154 mM NaCl, 2 mM KCl, 1 mM EDTA and 0.02% w/v NaN3). The supernatant was partially removed by three to four successive washes with centrifugation after each step. Platelets were collected after washing in 10 ml buffer A. The mononuclear cells RNA were extracted using QuickGene Mini80 (FUJIFILM, Japan) using a commercially available kit. The Superscript™ III First-Strand Synthesis System (Invitrogen, Carlsbad, California, USA) was used to obtain cDNA. The extracted cDNA samples were stored at −80°C for analysis at a later date.

#### Real-time PCR

Real-time PCR was performed using a Bio-Rad iCycler sequence detection system (Bio-Rad). The primers of the VLDL receptor, LDL receptor, and HMGCR are shown in Table 3. The reaction was added to 0.1 mL PCR strip tubes and were set by mixing 12.5 μl iQ™ SYBR® Green Supermix (BIO-RAD), with 1 μl cDNA, 0.5 μl primer (R), 0.5 μl primer (F), and 10.5 μl sterilized water. The plate was sealed and performed using a detector (iQ5 Multicolor Real-Time PCR Detection System). All volumes were normalized to β-actin expression (GenBank accession no. X00351) mRNA. The number of PCR cycles was defined as the mRNA levels and were quantified by use of the C_T_ value [34].

**Table 3:** **Primers used in real-time PCR analysis**

Gene	Primer sequence	Size (bp)	Tm (°C)
VLDL receptor	(F)ACCTCTGGCCAAATATGCAC	304	58.9
	(R)CACTCAGTCTTTGCAAACCTCC		
LDL receptor	(F)GCGTCCCTGTACAGATAGTGG	284	63.3
	(R)GCCACTCATACATACAACGG		
HMG-CoA reductase	(F)CCACGAACGCTCTTAGCTTTC	215	57.1
	(R)CTAAGAGGCTCTCCATGCTGC		
β-actin	(F)GGATGCAGAAGGAGATCACTG	90	
	(R)CGATCCACACGGAGTACTTG		

### Statistical analysis

The 6 hour total area under the TG concentration versus time curve (AUC) was calculated using the trapezium rule. The incremental area under the curve (iAUC) was calculated using the same method after correcting for fasting concentrations. The other metabolic parameters were calculated AUC and iAUC as previously described calculation of TG. All AUCs were compared among trials using a one-way analysis of variance (one-way ANOVA) with repeated measures. Plasma lipid, insulin concentrations and receptor mRNA expression were compared among trials and over time using a two-way ANOVA with repeated measures. Where appropriate post hoc pairwise comparisons were made using the Bonferroni method. Statistical significance was set at the 0.05 level of confidence. All results are expressed as means ± SEM.
